# A Genomic Island in *Salmonella enterica* ssp. *salamae* Provides New Insights on the Genealogy of the Locus of Enterocyte Effacement

**DOI:** 10.1371/journal.pone.0041615

**Published:** 2012-07-30

**Authors:** P. Scott Chandry, Simon Gladman, Sean C. Moore, Torsten Seemann, Keith A. Crandall, Narelle Fegan

**Affiliations:** 1 CSIRO Division of Animal, Food and Health Sciences, Werribee, Victoria, Australia; 2 Victorian Bioinformatics Consortium, Monash University, Clayton, Victoria, Australia; 3 Computational Biology Institute, George Washington University, Ashburn, Virginia, United States of America; Centre National de la Recherche Scientifique, France

## Abstract

The genomic island encoding the locus of enterocyte effacement (LEE) is an important virulence factor of the human pathogenic *Escherichia coli*. LEE typically encodes a type III secretion system (T3SS) and secreted effectors capable of forming attaching and effacing lesions. Although prominent in the pathogenic *E. coli* such as serotype O157:H7, LEE has also been detected in *Citrobacter rodentium, E. albertii,* and although not confirmed, it is likely to also be in *Shigella boydii.* Previous phylogenetic analysis of LEE indicated the genomic island was evolving through stepwise acquisition of various components. This study describes a new LEE region from two strains of *Salmonella enterica* subspecies *salamae* serovar Sofia along with a phylogenetic analysis of LEE that provides new insights into the likely evolution of this genomic island. The *Salmonella* LEE contains 36 of the 41 genes typically observed in LEE within a genomic island of 49, 371 bp that encodes a total of 54 genes. A phylogenetic analysis was performed on the entire T3SS and four T3SS genes (*escF*, *escJ, escN,* and *escV*) to elucidate the genealogy of LEE. Phylogenetic analysis inferred that the previously known LEE islands are members of a single lineage distinct from the new *Salmonella* LEE lineage. The previously known lineage of LEE diverged between islands found in *Citrobacter* and those in *Escherichia* and *Shigella.* Although recombination and horizontal gene transfer are important factors in the genealogy of most genomic islands, the phylogeny of the T3SS of LEE can be interpreted with a bifurcating tree. It seems likely that the LEE island entered the Enterobacteriaceae through horizontal gene transfer as a single unit, rather than as separate subsections, which was then subjected to the forces of both mutational change and recombination.

## Introduction

*Salmonella enterica* subspecies *salamae* serovar II 1,4,12,27:b:- (also referred to as serovar Sofia) is an infrequently detected serovar except for one niche, commercially produced broiler chickens in eastern Australia, in which it is the most frequently isolated *Salmonella* serovar (reviewed [1]). Unlike the cosmopolitan and more frequently isolated *S. enterica* subspecies *enterica* from which most pathogenic *Salmonella* are derived, the *salamae* subspecies generally has a more restricted distribution and is not frequently associated with virulence in humans or animals. *Salmonella* pathogenicity is often associated with intracellular invasion dependent upon functions encoded by a range of *Salmonella* pathogenicity islands (reviewed [2], [3]). Much of the focus of *Salmonella* research has been on members of the *S. enterica* subspecies *enterica* and includes 20 completed genome sequences (NCBI Microbial Genomes Database Nov. 2011) while only limited research has focused on the other five subspecies of *S. enterica* (*salamae, arizonae, diarizonae, houtenae,* and *indica*) and the other *Salmonella* species *S. bongori.* The genome of the second *Salmonella* species, *S. bongori* [4] has recently been completed while *S. enterica* subspecies *arizonae* (NC_010067) has been sequenced but not published and the genome sequences of several other subspecies are currently underway.

To gain a better understanding of the most commonly isolated *Salmonella* serovar from Australian poultry, a chicken carcass derived isolate was selected for genome sequencing. Although primarily isolated from poultry, small numbers of *S. enterica* subspecies *salamae* serovar Sofia are isolated from other niches including humans [5]. One of these atypical human derived isolates was also selected for genome sequencing. The genome sequence of the poultry derived *S. enterica* subspecies *salamae* serovar Sofia isolate is being taken to completion but during the first pass annotation of the draft assembly of these genomes a novel genomic island (GI) closely resembling the locus of enterocyte effacement (LEE) was detected. This discovery represents the first isolation of LEE from *Salmonella.*

The locus of enterocyte effacement is a collection of genes present in a variety of enteric organisms that has been demonstrated to play an important role in the virulence of human pathogenic *Escherichia coli* including enteropathogenic *E. coli* (EPEC), atypical enteropathogenic *E. coli* (ATEC), and enterhaemorrhagic *E. coli* (EHEC). LEE function is characterized by the formation of attaching and effacing (A/E) lesions after attachment of the *E. coli* cell to an enterocyte. LEE typically contains genes that encode a type III secretion system (T3SS), secreted effectors, regulatory molecules, chaperones, and accessory proteins required for the formation of the A/E lesion. The activity of the LEE island has been reviewed previously [6], [7] but can be briefly summarized as follows. Initial attachment of a bacterial cell to the host enterocyte by a bacterial cell surface adhesin is followed by penetration of the enterocyte cell membrane by the needle-like structure of the T3SS which then translocates effector proteins into the enterocyte. The key effector protein is the translocatable intimin receptor (Tir) that inserts into the enterocyte cell membrane and acts as an anchor to bind intimin (Eae) protein on the surface of the bacterial cell. This protein binding acts to lock together the enterocyte cell membrane and the outer membrane of the LEE expressing cell. In addition to its role in interacting with Eae, Tir also plays a role in polymerization of actin within the enterocyte to form the pedestal characteristic of the A/E lesion on which the bacterial cell sits.

The LEE island has been found in *E. coli*, *Citrobacter rodentium*, *Escherichia albertii,* and is also likely present in *Shigella boydii* based on the detection of the *eae* gene [8]. LEE is usually composed of 41 genes within a region of ∼35 kb in length. Nucleotide composition of LEE is ∼38% G+C which is substantially lower than the G+C content of the genomes of organisms known to harbor it (∼50%). Flanking sequences outside the 41 gene core of LEE are more variable in both gene content and nucleotide composition. Comparative analysis of flanking sequences by Müller et al. [9] demonstrated that previously described LEE islands ranged from those lacking any flanking sequences to those with large flanking regions encoding multiple genes and containing prophage remnants. Insertion of LEE into the genome generally occurs at one of three tRNA genes, *pheV, pheU,* or *selC*.

The LEE region has been well studied in *E. coli* and *Citrobacter rodentium* including a detailed analysis undertaken in *C. rodentium* to systematically create knockout mutants of every gene in LEE then characterize their effects on pathogenicity, T3SS function, and LEE gene expression [10]. No optimal *E. coli* LEE animal model exists but *C. rodentium* LEE is an effective model system for studying LEE activity because it is a natural mouse pathogen with a LEE island that has a high level of sequence identity to the LEE present in *E. coli*. These knockout experiments clearly demonstrated the requirement for the T3SS structure as well as several secreted effectors for the generation of the A/E lesion and for virulence. In addition, bioinformatic analysis of the genes of the LEE island were able to make further predictions on the function of several LEE genes lacking experimentally determined functions [11].

It is clear that horizontal gene transfer (HGT) plays an important role in the evolution of pathogenicity regions such as LEE [6]. LEE most likely entered the Enterobacteriaceae through HGT from an as yet unknown source. Previous analysis has demonstrated that most LEE genes are under purifying selection which is not surprising given the conservation of low G+C content and the high level of identity between the known LEE islands [12]. It is also clear that recombination has played an important role in the evolution of the LEE. T3SS are a prevalent pathogenicity mechanism throughout the Enterobacteriaceae [13] and the most well conserved subset of genes within LEE. This conservation of the T3SS genes has led to these genes being the basis for previous estimates of the genealogy of LEE [9]. Several authors have undertaken limited phylogenetic analyses of LEE and from this work it appeared that there were two lineages of LEE one of which was then subdivided into two major groups [9], [12].

In this study we describe a novel lineage of the LEE region present in two isolates of *S. enterica* subspecies *salamae* that has important implications for the genealogy of LEE. We present a detailed phylogenetic analysis of the entire T3SS of LEE as well as rooted single gene phylogenetic analyses of *escF*, *escJ, escN,* and *escV* to better understand the genealogy of LEE within the Enterobacteriaceae.

## Materials and Methods

### Ethics Statement

The human derived microbial sample (S1635) was collected originally published using the strain identifier MH76 [14]. This sample was derived from a strain stored at a pathology testing laboratory which was collected from a human with unspecified disease state and no specific collection date. Under the terms of the Australian National Statement on Ethical Conduct in Research Involving Humans and the associated legislation the National Health and Medical Research Council Act (1992) collection of a host de-identified, microorganism at the time of collection (prior to 1992) required no Human Research Ethics Committee approval. The chicken carcass derived microbial sample (S1296) was collected off the end of the production line at a licensed commercial poultry processing facility. This sample would therefore be regarded as food and did not require institutional review board or ethical approval.

### *Salmonella* Strains

*S. enterica* subspecies *salamae* serovar 1,4,12,27:b:- (also referred to as serovar Sofia) strains S1296 and S1635 were isolated in Australia from a chicken carcass and a human of unknown disease state respectively. The serovar designation follows standard conventions [15] where “1,4,12,27” refer to the O antigen types with underlined antigen types indicating seroconversion by phage lysogeny (so not necessarily present in all strains), “b” refers to the phase 1 H antigen (flagella), and the “-”indicates the absence of the phase 2 H antigen. These strains have different cell surface phenotypes and distinct *Xba*I restriction digestion patterns [16]. Strain S1296 was selected for genome sequence analysis because it was a typical representative of the poultry derived *S. enterica* subspecies *salamae.* Strain S1635 (also published as MH76 [14]), kindly provided by Dr Michael W. Heuzenroeder and Dr Ian L. Ross, Infectious Diseases Laboratories, Institute of Medical and Veterinary Science, Adelaide, South Australia was selected as representative of the infrequently isolated *S. enterica* subspecies *salamae* serovar Sofia derived from a non-poultry source.

### Genomic Island Sequence

Bacterial cultures were grown overnight in nutrient broth at 37°C prior to preparation of genomic DNA with (Qiagen DNeasy blood and tissue kit) following the manufacturer’s instructions for Gram-negative bacteria. Draft genomic DNA sequences for *S. enterica* subspecies *salamae* S1296 and S1635 were determined with combined Illumina 250 bp insert size paired-end sequencing (Micromon, Department of Microbiology, Monash University) and Roche paired-end pyrosequencing with 3 kb inserts (Australian Genome Research Facility). Roche pyrosequencing was assembled with Roche gsAssembler 2.4. The genomic islands and adjacent genome sequence described in this manuscript were located within one contig in both genomes. The integrity and accuracy of these sequences were assured by correcting homopolymer errors and polymorphisms by mapping Illumina paired-end sequence data onto the contigs using Nesoni v0.35 (www.bioinformatics.net.au). Absence of structural anomalies (e.g., large deletions, rearrangements, etc.) were screened by two methods. First, mapping the paired-end relationships of Illumina data with inGAP-sv [17] was used to detect improper paired-end fragment distances within the assembled sequence. The second method confirmed the integrity of the assembly by comparison of computer predicted *Nco*I restriction fragment sizes with restriction fragment sizes determined by optical mapping using MapSolver 3.2.0 (optical mapping and software supplied by OpGen Inc., USA). Sequences for the complete LEE genomic islands from both S1296 and S1635 have been submitted to Genbank (accession JQ747523 and JQ747540 respectively). The Genbank entries include the LEE flanking genes, *pheV*, and adjacent genomic genes *yqgA* and *ars.*

### Gene Selection

The source of all DNA sequences analyzed in this paper are listed in Table 1 with additional information provided in Table S1. Full strain designations are used in the body text of the manuscript while abbreviated taxon identifiers are used in the trees. Taxon identifiers utilize standard three letter genera – species abbreviations. For *E. coli strains* this is followed by O antigen type unless more than one of any given type is present in which case a strain identifier is appended. Non-*E. coli* strains utilize the three letter abbreviation combined with the strain identifier (key present in Table 1). Complete LEE regions present in the NCBI Genbank database at the commencement of this study were used for phylogenetic analysis. Organisms containing the LEE region for which a whole genome sequence was available were included in the housekeeping gene analysis along with several other genomes lacking the LEE region to provide a context for the tree as well as an outgroup. This tree was updated upon the release of the *Salmonella bongori* genome sequence [4]. The 17 housekeeping genes were selected based on housekeeping genes used in previous studies [18], [19] and included *accD, dcuA, galK, hemC, ilvE, ksgA, murD, oppB, pabB, pntB, polB, purB, rnc, rpoN, tesB, thrB,* and *trpB.* Accession numbers for *S. enterica* subspecies *salamae* S1296 and S1635 housekeeping genes are listed in Table S1.

**Table 1:** LEE and non-LEE containing strains used in this study.

Strains containing LEE	Genome sequence^a^	tRNA insertion point	Taxon Identifier	Accession Number
*E. coli* ATEC O119:H9:K61 0181-6/86		*selC*	Eco_O119	AJ633129
*E. coli* STEC O157:H7 Sakai^b^	+	*selC*	Eco_O157	NC_002695
*E. coli* O55:H7 CB9615	+	*selC*	Eco_O55	NC_013941
*E. coli* EPEC O127:H6 E2348/69	+	*selC*	Eco_O127	NC_011601
*E. coli* O111:H- 11128	+	*pheV*	Eco_O111	NC_013364
*E. coli* BSTEC O26:H 413/89-1		*pheU*	Eco_O26_413	AJ277443
*E. coli* O26:H11 11368	+	*pheU*	Eco_O26_11368	NC_013361
*E. coli* REPEC O15:H- 83/39		*pheU*	Eco_O15	AF453441
*E. coli* BSTEC O103:H2 RW1374		*pheV*	Eco_O103_RW1374	AJ303141
*E. coli* O103:H2 12009	+	*pheV*	Eco_O103_12009	NC_013353
*E. coli* ATEC O8:H- 3431-4/86^c^		*pheU*	Eco_O8	AJ633130
*E. albertii* TW07627	+	n/a	Eal_TW07627	NZ_ABKX00000000
*C. rodentium* DBS100		n/a	Cro_DBS100	AF311901
*C. rodentium* ICC168	+	n/a	Cro_ICC168	NC_013716
*S. enterica* subsp. *salamae* Sofia S1296	+	*pheV*	Sen_S1296	JQ747523
*S. enterica* subsp. *salamae* Sofia S1635	+	*pheV*	Sen_S1635	JQ747540
Strains used for housekeeping genes only				
*S. enterica* subsp. *enterica* Typhimurium LT2	+		Sen_STM	NC_003197
*S. enterica* subsp. *enterica* Typhi CT18	+		Sen_TY	NC_003198
*S. enterica* subsp. *arizonae* 62:z4,z23:– RSK2980	+		Sen_Ariz	NC_010067
*Vibrio cholerae* O1 biovar El Tor N16961	+		Vch_N16961	NC_002505 & NC_002506
*Salmonella bongori* NCTC 12419	+		Sbo_NCTC12419	NC_015761
*Shigella sonnei* Ss046	+		Sso_Ss046	NC_007384

a Completed or draft genome sequence available and used for housekeeping gene phylogeny.

b *E. coli* STEC O157:H7 str. Sakai is the representative strain for the other *E. coli* O157 LEE.

c Corrected likely sequence error that caused a frame shift mutation by deleting a C at position 208 in the CDS encoding *cesA.*

### Genomic Island Comparisons

The LEE core regions of the relevant genomic islands from *E. coli* O157:H7 Sakai (NC_002695) and *S. enterica* subspecies *salamae* S1296 (JQ747523) were manually extracted for generation of a linear comparison maps. A 38, 260 bp region was extracted from *S. enterica* subspecies *arizonae* RSK2980 (NC_010067) for comparison with the flanking region of the S1296 LEE island. Translated BLAST comparison (tblastx) and map generation were performed with Easyfig 2.1 [20] with minimum length of BLAST hits set to 20 and maximum E-value set to 0.001.

### Phylogenetic Analysis

The nucleotide sequence of each gene present in the *Salmonella* LEE (Table 2) and all of the housekeeping genes were aligned to the appropriate ortholog from the known LEE regions or genomes listed in Table 1. Single gene multisequence alignments were created with Mega 5.05 [21] by translating DNA sequences into amino acids then aligning with ClustalW and Muscle followed by conversion back to DNA sequence. Gaps and poorly aligned regions were removed with Gblocks [22] using default settings for codon based alignments and a custom script was used to concatenate all LEE genes or just the T3SS genes into sequence matrices. All matrices were tested for discrepancies in the nucleotide composition using SeqVis 1.5 [23] (Figure S1). Evolutionary substitution models were analyzed using jModelTest 0.1.1 [24], [25]. Genetic diversity (θ) per site was estimated for Gblocks curated alignments using the Watterson estimate [26] assuming infinite sites as implemented in DNAsp ver. 5.10.01 [27] for every gene in LEE. Standard deviations of θ were calculated allowing for free recombination. A sliding window analysis of θ was conducted for the T3SS alignment with and without *Salmonella* sequences present using a step size of one base and a window size of 100 bases.

**Table 2:** Genes in the SESS LEE genomic island.

Locus Tag	MW	Residues	% G+C	Gene^a^	Product^b^	T3SS^c^	θ+Sal^d^	θ-Sal^e^
SESS1296_03597	n/a	n/a	n/a	*pheV*	tRNA Phe			
SESS1296_03598	48,082	423	54.3		Bacteriophage integrase			
SESS1296_03599	25,687	230	46.7		Putative phosphatase/phosphohexomutase			
SESS1296_03600	18,902	163	44.0		Predicted carbamoyltransferase			
SESS1296_03601	43,931	387	45.3		Putative carbamoyltransferase			
SESS1296_03602	8,724	75	42.7		Hypothetical protein			
SESS1296_03603	32,476	292	51.5		Putative transcriptional regulator			
SESS1296_03604	16,255	149	47.9		Hypothetical protein			
SESS1296_03605	14,066	116	40.2		Hypothetical protein			
SESS1296_03606	18,208	162	41.6		Putative HCP familysecreted effector			
SESS1296_03607	9,022	80	45.8		Hypothetical protein			
SESS1296_03608	6,862	63	51.9		Hypothetical protein			
SESS1296_03609	14,850	134	45.0		Hypothetical protein			
SESS1296_03610	41,393	370	58.0		Putative integrase			
SESS1296_03611	9,992	91	55.3		Hypothetical protein			
SESS1296_03612	163,610	1398	35.6		Conserved hypotheticalprotein			
SESS1296_03613	79,817	706	49.9		Putative transcriptional regulator			
SESS1296_03615	9,631	81	43.2		Putative phage integrase			
SESS1296_03616	26,596	232	31.6		Putative DNA-bindingtranscriptional activatorof the LuxR/FixJ family			
SESS1296_03617	9,690	87	36.4	*orf29* f	T3SS	+	0.231 (0.033)	0.143 (0.027)
SESS1296_03618	8,098	74	35.6	*escF*	Major needle component	+	0.15 (0.027)	0.141 (0.026)
SESS1296_03619	16,000	138	35.5	*cesD2*	Chaperone for EspD		0.126 (0.017)	0.051 (0.012)
SESS1296_03620	35,342	331	44.6	*espB*	Translocator		0.377 (0.024)	0.285 (0.02)
SESS1296_03621	39,266	375	45.2	*espD*	Translocator		0.256 (0.017)	0.197 (0.015)
SESS1296_03622	20,381	191	40.8	*espA*	Translocator		0.198 (0.02)	0.183 (0.019)
SESS1296_03623	39,792	347	32.0	*sepL*	Switches translocator/effector secretion		0.134 (0.011)	0.059 (0.008)
SESS1296_03624	45,390	409	36.3	*escD*	T3SS component	+	0.157 (0.011)	0.043 (0.006)
SESS1296_03625	108,364	1015	44.8	*eae*	Adhesin (intimin)		0.253 (0.011)	0.176 (0.01)
SESS1296_03626	17,701	157	35.0	*cesT*	Chaperone for Tir		0.065 (0.011)	0.028 (0.007)
SESS1296_03627	56,721	544	49.6	*tir*	Secreted effector		0.436 (0.02)	0.323 (0.017)
SESS1296_03628	14,901	129	41.3	*ler*	Positive regulator		0.162 (0.026)	0.079 (0.019)
SESS1296_03629	9,835	85	33.3	*escE*	T3SS component	+	0.327 (0.047)	0.066 (0.021)
SESS1296_03630	12,062	103	33.0	*cesA*	Chaperone for EspAand EspB		0.267 (0.033)	0.102 (0.021)
SESS1296_03631	23,339	200	33.2	*orf4* f	T3SS component	+	0.195 (0.019)	0.059 (0.01)
SESS1296_03632	26,838	228	33.9	*escL*	T3SS component	+	0.304 (0.023)	0.15 (0.016)
SESS1296_03633	24,166	218	32.1	*escR*	T3SS component	+	0.074 (0.01)	0.033 (0.007)
SESS1296_03634	9,722	88	34.1	*escS*	T3SS component	+	0.063 (0.016)	0.027 (0.012)
SESS1296_03635	28,644	259	33.8	*escT*	T3SS component	+	0.112 (0.012)	0.04 (0.007)
SESS1296_03636	39,183	346	33.0	*escU*	T3SS component	+	0.102 (0.01)	0.044 (0.007)
SESS1296_03637	17,167	154	29.4	*rorf3 (etgA)*	Predicted lytictransglycosylase		0.156 (0.02)	0.059 (0.013)
SESS1296_03638	12,598	114	30.7	*grIR*	Negative regulator		0.208 (0.024)	0.084 (0.017)
SESS1296_03639	15,981	135	35.3	*grIA*	Positive regulator		0.14 (0.019)	0.049 (0.01)
SESS1296_03640	17,398	151	36.4	*cesD*	Chaperone for EspD		0.077 (0.014)	0.031 (0.009)
SESS1296_03641	56,789	519	35.6	*escC*	T3SS component	+	0.072 (0.007)	0.028 (0.005)
SESS1296_03642	17,436	152	31.6	*sepD*	Switches translocator/effector secretion		0.181 (0.021)	0.074 (0.015)
SESS1296_03643	20,471	188	33.5	*escJ*	T3SS component	+	0.122 (0.015)	0.029 (0.008)
SESS1296_03644	12,866	120	37.8	*rorf8 (escI)*	T3SS component	+	0.165 (0.023)	0.098 (0.016)
SESS1296_03645	8,586	92	49.3	*sepZ (espZ)*	Secreted effector		0.495 (0.055)	0.353 (0.045)
SESS1296_03646	13,909	118	26.3	*orf12* f	T3SS component	+	0.215 (0.026)	0.046 (0.013)
SESS1296_03647	75,251	676	36.8	*escV*	T3SS component	+	0.078 (0.006)	0.028 (0.004)
SESS1296_03648	48,475	444	42.0	*escN*	T3SS ATPAse	+	0.086 (0.008)	0.034 (0.005)
SESS1296_03649	14,925	127	30.2	*orf15* f	T3SS component	+	0.262 (0.028)	0.063 (0.013)
SESS1296_03650	16,829	141	30.5	*orf16* f	Secretion of translocators		0.37 (0.034)	0.15 (0.022)
SESS1296_03651	33,795	296	33.3	*sepQ (escQ)*	T3SS component	+	0.302 (0.02)	0.125 (0.013)
SESS1296_03652	28,680	261	45.6	*espH*	Secreted effector		0.386 (0.035)	0.277 (0.029)

a Gene names modified from Table 1 in Pallen et al. [11] with alternative names.

b Product names derived from annotation of SESS1296 and modified from Table 1 in [11].

c Structural components of the T3SS are indicated with (+).

d Diversity (θ) for the listed LEE gene from all alignments including the two *Salmonella* LEE with standard deviations listed in parentheses.

e Diversity (θ) for the listed LEE gene from all alignments excluding the two *Salmonella* LEE with standard deviations listed in parentheses.

f *Salmonella* variants of LEE genes with “orf” designations utilize locus tags as gene names.

Bayesian Inference (BI) was performed using a multiprocessor implementation of MrBayes 3.2 [28]. In line with the results of jModelTest, the General Time Reversible model (nst = 6) was used for all analyses with GTR+G used for housekeeping genes and GTR+I+G implemented for the T3SS matrix as well as the *escF*, *escJ, escN,* and *escV* alignments. Data were partitioned by the three positions in the codon and BI analyses were run using the 4by4 nucleotide model for 10×10^6^ generations. Convergence metrics were assessed using Tracer 1.5 [29] to assure that the samples used for burnin excluded data prior to achieving a stable plateau in the ln likelihood values and that sufficient sampling was completed. The majority rule tree (and all other trees in this manscript) was then rendered with FigTree 1.3.1 [30] and Mega 5.05 [21] with posterior probabilities converted to a percentage. BI analyses were run a minimum of three times for each alignment to ensure that the resulting tree was reproducible.

Maximum likelihood (ML) analysis was performed using Garli [31] with a GTR (nst = 6) based codon model. Garli was selected for ML analysis because of its capacity to use a codon model and rigorous likelihood searching methods. Codon frequencies were set to F3×4 and a single dN/dS parameter was used. Individual analyses were terminated when the ln likelihood value was not improved after 3000 generations. Garli does not perform traditional bootstrapping [32], instead 500 independent runs were used as “bootstrap replicates” and summarized with SumTrees 3.3.1 [33]. Replicate runs of Garli were limited to 500 iterations due to the protracted time required for each iteration. Bootstrap results were then expressed as a percentage.

### Recombination and Selection Estimation

#### Recombination testing was undertaken with PHIPack

(http://www.maths.otago.ac.nz/~dbryant/software.html) which implements the pairwise homoplasy index (Φ_ω_) [34], maximum χ^2^ [35], and neighbour similarity score (NSS) [36] using 5000 permutations. The impact of recombination on phylogeny was gauged using ClonalFrame [37] which uses a Bayesian inference method to determine phylogeny accounting for both spontaneous mutations and recombination events. ClonalFrame was run with standard settings that estimated the rate of recombination as well as being run with recombination fixed at none. Single runs of ClonalFrame included 400,000 iterations after which 200,000 iterations were excluded for burn-in. The results from ClonalFrame were consistent for 4 replicate runs with all runs yielding identical trees. ClonalFrame was also used to estimate the ratio of the frequency of recombination to mutation (ρ/θ). Individual gene alignments were combined into a ClonalFrame readable file using a custom script.

Calculation of the site by site variation in the ratio of non-synonymous to synonymous nucleotide substitution rates (dN/dS) was estimated using the fixed effects likelihood method (FEL) [38] as implemented in the web based Datamonkey software suite [39]. Following selection of the optimal evolutionary model (012232) by Datamonkey codons were reported to be positively or negatively selected at a significance level of 0.05. The code string 012232 is a reversible model of nucleotide substitution indicating the number of distinct parameters for the relative rates of A↔C, A↔G, A↔T, C↔G, C↔T, G↔T, respectively. We chose the FEL approach because it directly estimates non-synonymous and synonymous substitution rates across sites and has been shown to better capture the patterns of rate variation when compared to counting methods and random effects models and does not suffer from a relatively higher false positive rate of the random effects models when sequence numbers are low, as in our data [38].

## Results

### General Description of *Salmonlla*-LEE Genomic Island

**Figure 1:**
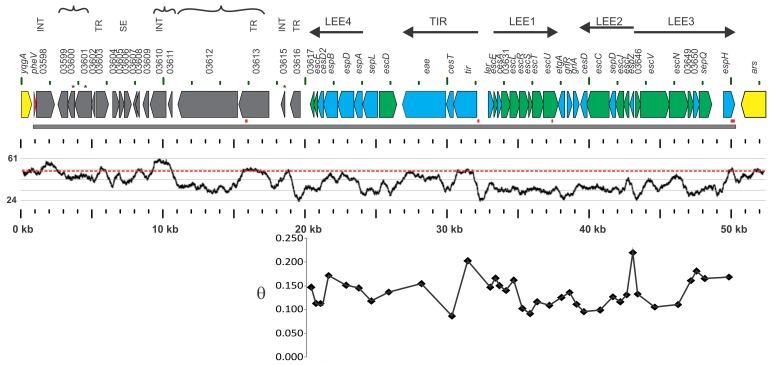
Schematic of the genomic island containing LEE. The extent of the genomic island is marked by the solid gray line and the bounding genomic genes outside the genomic island are marked in yellow. Genes that are not part of the LEE region are marked in grey. Genes that form the T3SS are shown in green, the *pheV* tRNA insertion point is marked by a small red arrow with the remainder of the LEE region in blue. Redundant text from the locus tags has been removed to simplify the figure. The full locus tag of those genes designated with a 4 digit number would read SESS1296_0XXXX. The operon structure of the LEE region is indicated by black arrows. Gene functions are abbreviated, putative integrase (INT), putative transcriptional regulator (TR), putative secreted effector (SE). Groups of genes for which homologs co-occur in other organisms are indicated by curly brackets. Possible pseudogenes are marked with (*). G+C content calculated with a 100 bp window is depicted by the graph below the diagram with maximum (upper line = 61%) and minimum (lower line = 24%) values designated. Typical G+C content of 52% for *Salmonella* genomes is depicted by the dashed red line. Four tandem repeat regions are depicted by small red boxes. A plot of genetic diversity (θ) calculated for each LEE gene using a Watterson estimate is represented as a spot below the relevant gene.

The locus of enterocyte effacement was detected in the sequences of the two *S. enterica* subspecies *salamae* strains (S1296 and S1635) (Table 1). It is inserted at the 3' end of tRNA^Phe^ (*pheV*) adjacent to the *yqgA* gene upstream and a putative arylsulfatase (*ars*) downstream (Figure 1). The total length of the genomic island in S1296 from the 3' end of *pheV* to the tandem repeat region at the 3' end of *espH* is 49, 371 bp. A total of 54 open reading frames (ORFs) are present within the GI, 36 of which encode the LEE core. A large flanking region encoding 18 ORFs is present at the 5' end of LEE in both *Salmonella* strains S1296 and S1635. The G+C content of the entire GI is 40.0% while the G+C content of the LEE encoding portion is 37.4% with only six genes in the entire GI at or above the typical *Salmonella* G+C content of ∼52% (Figure 1 and Table 2). There are four tandem repeats within the SESS LEE of sizes 42, 36, 21, and 114 bp, with copy numbers of 3.8, 2.0, 1.9, and 2.3, respectively. No repeat of the tRNA^Phe^ was detected at the 3' end of the genomic island but a partial repeat of this tRNA was detected between the flanking sequences and core LEE gene SESS_03617.

### Flanking Region of LEE

The majority of the 18 ORFs within the flanking region of the *S. enterica* subspecies *salamae* serovar Sofia LEE (SESS LEE) island could not be assigned a function by homology transfer. Both strains S1296 and S1635 have the same complement of genes in the LEE flanking regions which vary between the strains primarily by a small number of single nucleotide polymorphisms. The first gene in the LEE flanking region (SESS1296_03598), downstream from *pheV* is a CP4 type integrase. Another integrase (SESS1296_03610) is present near the midpoint of the flanking region and an amino terminus remnant of another integrase (SESS1296_03615) is present near the 3' end of the flanking region. Approximately, 47% of the SESS LEE flanking region shares substantial homology with two clusters of genes from a putative genomic island inserted at a tRNA^Phe^ in *S. enterica* subspecies *arizonae* RSK2980 (SARI) (Figure S2). All the genes in the SARI genomic island are annotated as hypothetical, but adjacent to the tRNA insertion point is a putative integrase as well as an integrase remnant downstream. Two of the putative integrases share identity with homologs in the SARI genomic island as well as the two largest ORFs in the SESS LEE flanking region (SESS1296_03612 and SESS1296_03613), one of which is a transcriptional regulator. The region of the SARI genome sharing identity with the trasncriptional regulator does not encode a single ORF but rather several smaller ORFs with identity to SESS1296_03613.

In addition to the identity between the SESS LEE and a genomic island in SARI two clusters of ORFs appear to have common origins based on co-occurrence in other organisms detected in the BLAST search results (Figure 1). The first cluster of ORFs spanning SESS1296_03599 to SESS1296_03601 share a high level of identity with sequences found in other proteobacteria including several strains of *Pseudomonas aeruginosa* as well as *Marinobacter* and *Methylobacter*. Two of the ORFs in this cluster (SESS1296_03600 and SESS1296_03601) are the likely carboxyl-terminus and amino-terminus of a possible pseudogene resulting from a frame shift mutation in a putative carbomyl transferase. This putative pseudogene is adjacent to and co-occurs with a putative phosphotase/phosphohexomutase. The second cluster of ORFs for which homolgs co-occur in other organisms includes a putative integrase (SESS1296_03610) and a hypothetical protein (SESS1296_03611). These ORFs co-occur in other *S. enterica* including serovar Weltevreden and in *Citrobacter youngae* ATCC 29220.

As stated previously, only a limited number of ORFs could be assigned a function based on sequence identity. Putative transcriptional regulatory proteins are encoded by three of the ORFs but no regulatory elements could be directly assigned to these ORFs. The transcriptional regulator proximal to the core LEE region in S1635 (SESS1635_03831 an ortholog of SESS1296_03616) contains a 14 amino acid in-frame internal deletion when compared to the orthologous protein in S1296. Besides those functionally assigned genes discussed above, SESS1296_03606 was identified as a putative HCP family secreted effector because of high level of identity (∼75%) to comparably annotated proteins in other Enterobacteriales, but the function of this protein or export by a type VI secretion system are both uncertain.

### *Salmonella*-LEE Region Overview

**Figure 2:**
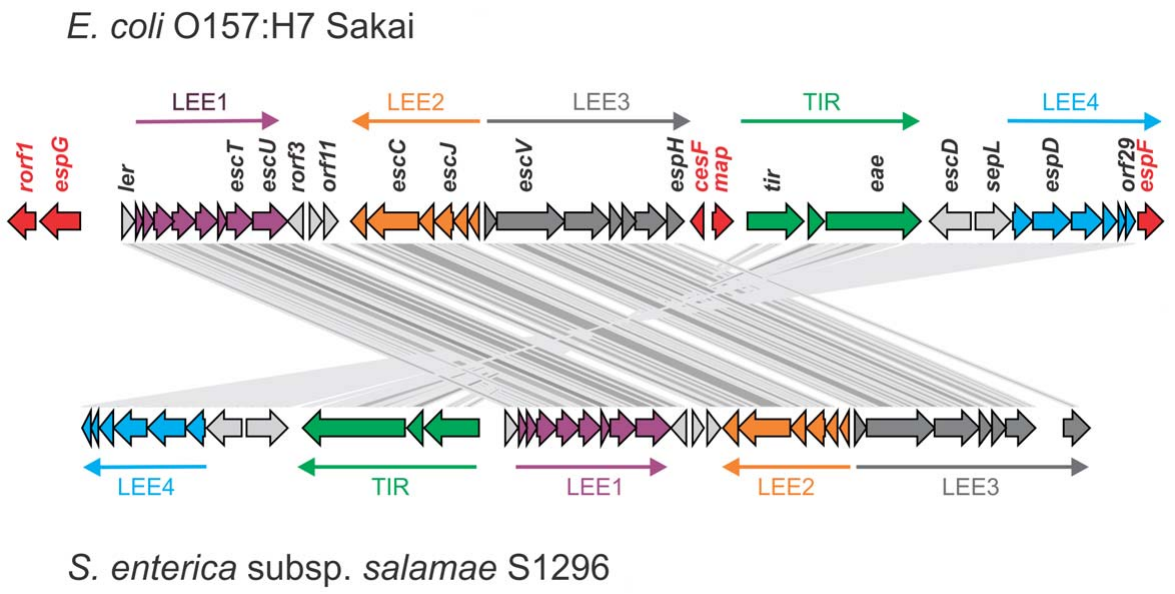
Comparison of the operon arrangements between SESS LEE and *E. coli* LEE. Core LEE genes from *E. coli* O157:H7 Sakai and *Salmonella* are connected by lines indicative of segments that match in a tblastx comparison. Genes and arrows indicating the extent of the LEE operons are colored to facilitate comparison. Genes outside the operons are colored gray and those genes present in *E. coli* but absent in *Salmonella* are colored red. Filled arrows are drawn to scale and accurately positioned based on the genome sequences they depict.

The SESS LEE encodes the majority of the genes (36 of the standard 41 genes) orthologous to the typical *Escherichia* and *Citrobacter* LEE and therefore the homology extends beyond the T3SS. The insertion of SESS LEE at *pheV* matches one of the three previously observed tRNA insertion points. Genes typically present in *Escherichia* and *C. rodentium* LEE are organized into five operons and this arrangement has been largely conserved in the SESS LEE (Figure 2). Although the orthologous genes are arranged in the appropriate operons, these operons have been rearranged such that operons TIR and LEE4 were reverse complemented and moved to the 5¢ end of LEE1, rather than the more typical arrangement at the 3' end of LEE3. The putative recombination junctions between the operons are the regions where the five genes typically seen in other LEE regions were lost in the SESS LEE region. These genes include *espG* and *rorf1* normally upstream of LEE1, *cesF* and *map* upstream of the TIR operon, and *espF* downstream of LEE4. Two tandem repeats also occur near the putative recombination junctions with one repeat 54 bases upstream of *tir* (36 bp period size and copy number of 2) and a second repeat within the 3' end of *espH* (114 bp period size and copy number of 2.3). There was insufficient similarity between intergenic regions of SESS LEE and the other known LEE to infer specific recombination break points.

The level of genetic diversity (θ) was calculated for every individual gene alignment with and without the SESS LEE included in the alignments (Table 2). A comparison of θ for the 36 LEE gene alignments lacking the SESS LEE demonstrated a variable but generally low level of diversity (median θ = 0.0634, mean θ = 0.074, standard deviation 0.033). This low level of diversity has been observed in previous studies of *E. coli* and *C. rodentium* LEE [12]. As expected, proteins not part of the T3SS structure such as *espZ, tir, espA, espB,* and *espH* had the highest levels of diversity. Addition of SESS LEE genes to the alignments resulted in a substantive increase in diversity but this increase was proportional with most genes roughly doubling in diversity (median θ = 0.134, mean θ = 0.136, standard deviation 0.030).

### Phylogeny of the SESS LEE Type III Secretion System

The evolutionary constraint of a functional structure and the previously observed low levels of genetic diversity [9], [12] make the T3SS an attractive target for phylogenetic analysis. Therefore, to understand the genealogical relationship between the SESS LEE and those from *E. coli*, *E. albertii,* and *C. rodentium*, the coding sequences of T3SS genes were used to generate a concatenated gene alignment matrix (Table 2). A concatenated gene matrix was selected as the phylogenetic method because of the limited genetic diversity between many of the taxa. Genetic diversity analysis calculated with and without the SESS LEE genes demonstrated a consistent difference of approximately θ = 0.065 across all of the genes of the T3SS matrix (Table 2 and Figure S3). The *escF* gene stands out from the other T3SS genes in having the least change in diversity raising it by only θ = 0.018 upon addition of SESS genes to the alignment matrix.

**Figure 3:**
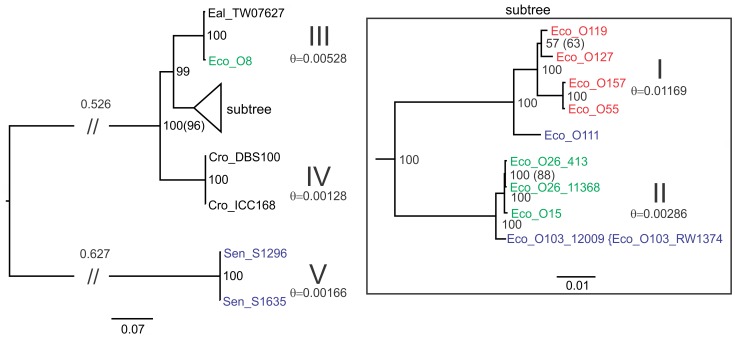
Phylograms of the consensus BI tree for the matrix of LEE T3SS genes. All diagrams are presented with long branches truncated (//) and the branch length indicated above. The subtree in the box represents BI branching on a different scale from the larger tree. Taxa are colored based upon the tRNA insertion point with *selC* in red, *pheV* in blue, *pheU* in green, and black for all other insertion points. Clades are indicated by large Roman numerals above calculated level of genetic diversity (θ) for all the members of the clade. The BI tree is midpoint rooted with posterior probability values represented as a percentage at branch points. ML analysis yielded the same branching (although differential branch lengths) and ML percentage bootstrap values are presented in parentheses () when this value differed from the percent posterior probability. Taxa enclosed by braces had identical sequences to the adjacent taxa and were therefore removed from the phylogenetic analysis.

Phylogenetic analysis of the LEE T3SS was determined by Bayesian inference (BI) and results presented as a midpoint rooted tree (Figure 3). For comparison, the phylogeny was also calculated by maximum likelihood (ML) which yielded the same branching pattern. The BI consensus tree demonstrates substantial divergence between the SESS LEE region and the other previously known enterobacterial LEE with high posterior probabilities supporting most of the branching patterns. Both BI and ML analysis suggest the relationship between *E. coli* O157:H7 Sakai/O55:H7 CB9615, *E. coli* O119:H9:K61 0181-6/86, and O127:H6 E2348/69 is unclear. Overall, this tree splits the LEE T3SS into five clades (Figure 3) with clades I-IV including the previously described LEE forming a distinctive lineage from the SESS LEE. The level of genetic diversity within these clades is generally low with clade I having the highest level of diversity due to the difference between *E. coli* O111:H- 11128 and the other members of this clade. Calculation of diversity within clade I excluding *E. coli* O111:H- 11128 yields è = 0.0068. The level of diversity between the two SESS LEE variants is greater than between the two *Citrobacter* variants in clade IV and about half that of the LEE T3SS in clade II. LEE T3SS regions derived from *C. rodentium* clearly segregate as separate lineage from the *Escherichia* which in turn diverges between *E. albertii* TW07627/*E. coli* O8:H- 3431-4/86 from all the other *Escherichia.* The remaining *E. coli* LEE T3SS genes split into two clades that generally correlate with the tRNA insertion point. The taxa within clade II diverge into two clusters that concur with the *pheU* and *pheV* tRNA insertion points while taxa in clade I diverge between *selC* and *pheV* tRNA insertion points.

**Figure 4:**
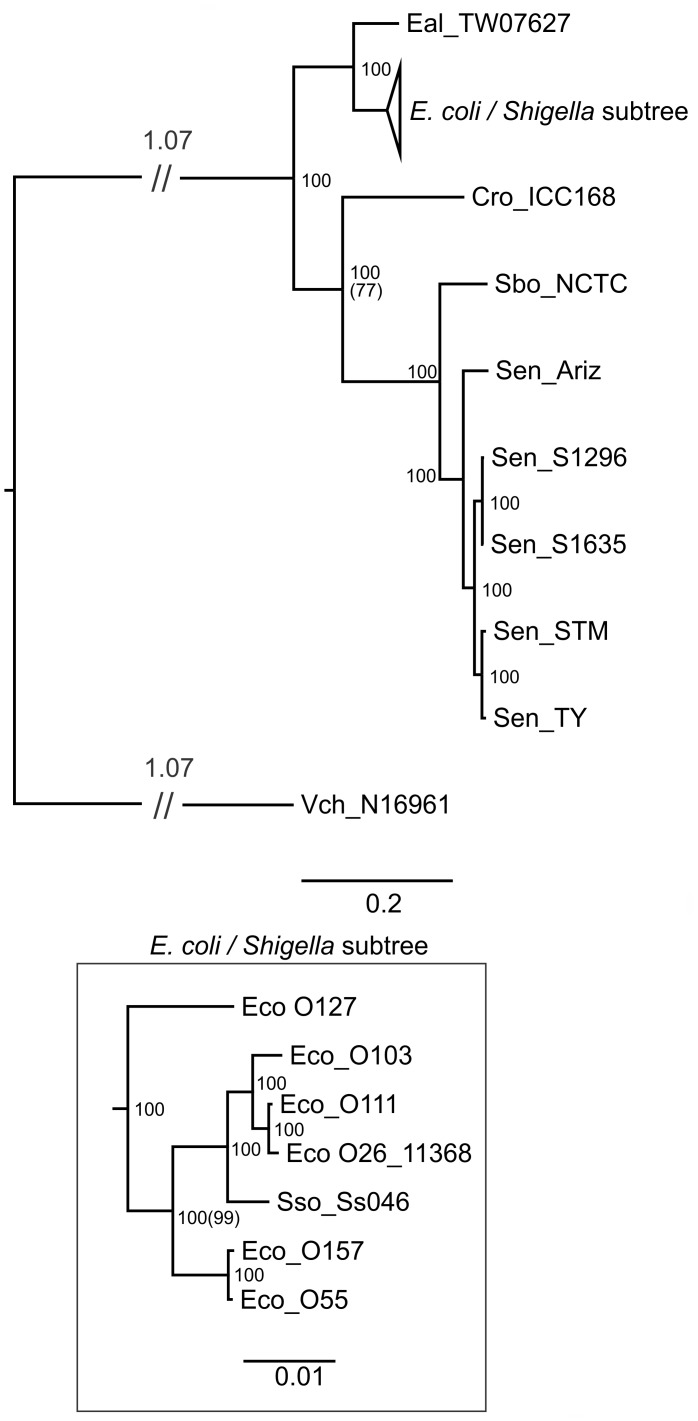
BI tree for the matrix of housekeeping genes. All diagrams are presented with long branches truncated (//) and the branch length indicated above. ML analysis yielded the same branching (although differential branch lengths) with ML percentage bootstrap values presented in parentheses () when this value differed from the percent posterior probability. The housekeeping genes from those taxa with LEE regions present in the Genbank database but lacking associated genome sequence data were not analyzed and therefore are not depicted in tree.

Comparison with the housekeeping gene tree (Figure 4) indicates that the genealogy derived from the housekeeping genes is substantively different from that observed for the LEE T3SS genes. The rooted housekeeping gene tree demonstrates that the lineage including *Salmonella/Citrobacter* and the *Escherichia* lineage diverged from a common ancestor some time ago. In contrast to the housekeeping gene tree, it is clear that the *C. rodentium* LEE T3SS is much closer to that found in *Escherichia* than it is to *Salmonella.* In addition, the housekeeping gene phylogeny suggests that the relationship between *E. coli* strains containing LEE bears little resemblance to the phylogeny of the LEE T3SS region itself (Figure 3). For example, *E. coli* O127:H6 E2348/69 forms a separate lineage of *E. coli* yet the LEE T3SS present in this strain is a member of clade I. Only the close relationship between *E. coli* O157:H7 Sakai and O55:H7 CB9615 is consistent between both housekeeping and LEE T3SS trees.

**Figure 5:**
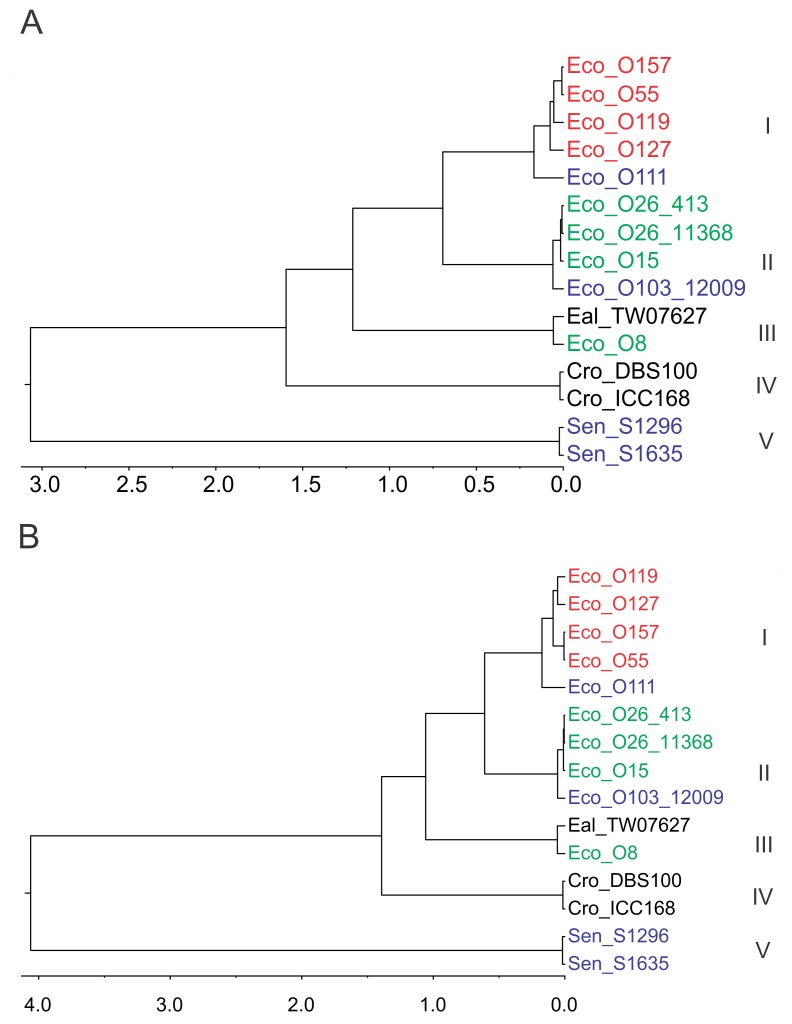
A 95% majority rule tree estimated for the T3SS genes with ClonalFrame allowing the program to determine the effect of recombination (A) or fixing the recombination rate at zero (B). Clades numbered I-V are indicated next to respective taxa. Taxa are colored based upon the tRNA insertion point with *selC* in red, *pheV* in blue, *pheU* in green, and black for all other insertion points. The scale is time in coalescent units.

The likelihood that recombination may be affecting the phylogenetic predictions for the T3SS data was examined with a combination of statistical tests for recombination (Table 3). Tests using Φ_ω_, maximum χ^2^, and NSS indicated that the null hypothesis of no recombination was rejected for all methods. Therefore, ClonalFrame, a Bayesian inference method to reconstruct the clonal relationship partitioned by individual genes was run assuming no recombination or permitting the program to estimate the recombination parameters from the data set (Figure 5). The resultant 95% majority rule tree estimating recombination matched most of the branching of the BI tree determined using Mr. Bayes 3.2 but varied in the arrangement of those taxa in clade I (those LEE inserted at *selC*). An alternative tree estimated by ClonalFrame assuming no recombination yielded the same branching pattern as observed for both BI and ML analysis. Estimation of the ratio of the frequency of recombination to mutation (ρ/θ) was 0.0166 (95% confidence interval [0.0104–0.024]) indicating that polymorphisms due to mutation exceeded those due to recombination.

**Table 3:** Results of statistical tests for recombination.

Alignment	Ö_ù_ a, b	Max ÷^2^	NSS
T3SS genes^c^	0.000	0.000	0.000
*escF* c	0.841	0.894	0.137
*escJ* c	0.044	0.783	0.764
*escN* c	0.000	0.001	0.0052
*escV* c	0.339	0.191	0.013

a P-value calculated with permutation test (5000 iterations).

b Window size was set to 100.

c *Salmonella* sequences removed from alignments prior to calculation.

Calculation of non-synonymous/synonymous (dN/dS) ratios for all of the codons in the T3SS matrix was used to determine if these genes were under selective evolutionary pressure. Using the FEL method 989 codons of 4420 were found to be under negative selective pressure while only three codons were found to be under positive selection. A graph of normalized dN/dS ratios for the T3SS alignment matrix is provided in the supporting information (Figure S3) reiterates this result and confirms previous observations that the genes composing the T3SS are under negative selective pressure [12].

### Single Gene Trees

**Figure 6:**
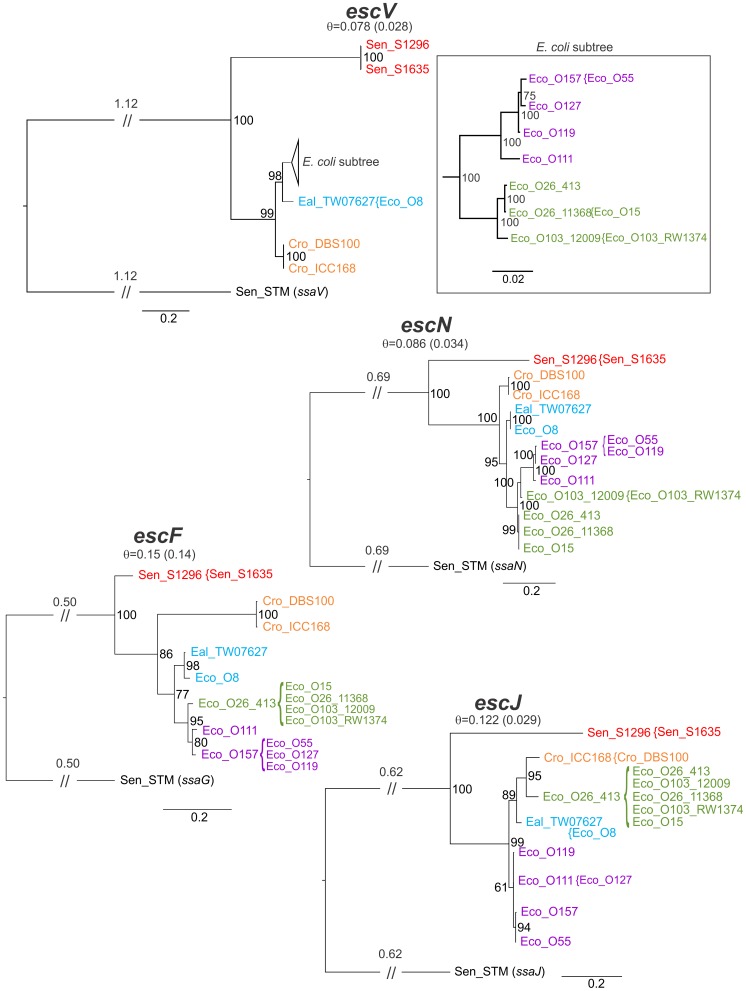
Rooted phylogenies for T3SS genes *escV, escN, escF,* and *escJ*. BI trees for the four T3SS genes described in Castillo et al., [**12**] are depicted with long branches truncated (//) and the total branch length indicated above. The subtree in the box represents BI branching on a different scale from the larger trees. Taxa are colored by presence in the clades described in Figure 3 and 5 to facilitate comparison between trees (clade I – purple, clade II – green, clade III – blue, clade IV – orange, clade V – red). Taxa enclosed by braces had identical sequences to the adjacent taxa and were therefore removed from the phylogenetic analysis. Diversity values (è) below gene names are calculated excluding the outgroup and those calculations also excluding the SESS LEE genes are in parentheses. Numerals at tree junctions are the percentage posterior probability.

The genealogy of the T3SS of LEE was examined by BI of a concatenated matrix of genes lacking an outgroup so it was presented as a midpoint rooted tree. Although this data set did not appear to be amenable to analysis by congruence of single gene trees, phylogenies calculated with an outgroup do provide insights on the order of descent. Therefore, to better understand the genealogy of the T3SS region of LEE, rooted phylogenies were prepared for *escV, escF, escN,* and *escJ* (Figure 6). These were the same four LEE T3SS genes previously analyzed by Castillo et al., [12]. Paralogs for each of the selected LEE T3SS genes were present in a well conserved T3SS common to most *S. enterica.*

The *escV* gene is a well-conserved structural component of the T3SS that has been used previously for phylogenetic comparison among LEE regions [9], [12]. A paralog of *escV* which is conserved in most *S. enterica* subspecies *enterica, ssaV* was selected to serve as an outgroup for the *escV* phylogeny. This single gene alignment contained several duplicate sequences which were removed, resulting in a final alignment containing 13 taxa and the lowest level of diversity of any of the single gene phylogenies. The resulting BI rooted tree (Figure 6) replicates the arrangement of taxa observed for the matrix of LEE T3SS genes with clade I poorly resolved. Contrary to the arrangement observed for the T3SS, the rooted phylogeny indicates that *escV* of *E. coli* O119:H9:K61 0181-6/86 split from a common ancestor prior to the split between the *E. coli* O157:H7 Sakai and O127:H6 E2348/69. The *escV* genes from the SESS LEE strains are the sole members of a second lineage in the *escV* genealogy. The other branch has a line of descent splitting first between *C. rodentium* and *Escherichia* then splitting the *Escherichia* between *E. albertii* TW07627*/E. coli* O8:H- 3431-4/86 and the remainder of the *E. coli*. Possible effects of recombination within the *escV* phylogeny were tested statistically as for T3SS matrix (Table 3). Tests using Φ_ω_ and max χ^2^ did not detect recombination in the *escV* alignment but the NSS test detected recombination. Given the propensity for NSS to yield a higher level of false positive results on some data sets than the other methods it seems likely that recombination is not a significant factor in the phylogeny of *escV* [34].

The single gene BI phylogeny containing a paralog of *escN* (*Salmonella* gene *ssaN*) resulted in 13 taxa after the removal of duplicate sequences. This phylogeny had a similar level of diversity and showed a similar line of descent to that observed for *escV.* There was one substantial difference at the point of divergence between the taxa in clades I and II. In this case *E. coli* O103:H2 12009 (and duplicate *E. coli* O103:H2 RW1374) shares a common ancestor with clade I taxa. The three statistical tests for recombination rejected the null hypothesis for all methods indicating that recombination was detected in the *escN* gene alignment (Table 3).

The *Salmonella* T3SS gene *ssaG* was selected as the appropriate outgroup for phylogenetic analysis of *escF.* This gene demonstrates the highest level of diversity of the four individual genes analyzed but interestingly has the least difference between the SESS *escF* and the other previously known *escF* genes. Given this level of diversity it is surprising that those taxa in clades I and II have very little diversity with all members of clade II identical and within clade I only *E. coli* O111:H- 11128 varies from the other taxa. Examination of the phylogeny of the 9 taxa remaining after duplicates were removed showed that the order of descent depicted in this gene tree reiterates the LEE T3SS phylogeny. Statistical tests did not detect recombination by any of three methods employed (Table 3).

The single gene BI phylogeny containing a paralog of *escJ* (*Salmonella* gene *ssaJ*) had 9 taxa after removal of duplicates and varied considerably from the branching observed for the other single gene phylogenies and the LEE T3SS. Except for the split between SESS *escJ* and other known *escJ* this tree has a different genealogy. The *escJ* phylogeny for the non-SESS taxa has a common ancestor diverging into two sister lineages one containing a polytomy between *E. coli* O119:H9:K61 0181-6/86, *E. coli* O111 11128, and *E. coli* O157:H7 Sakai/O55:H7 CB9615. The other lineage has no similarity to previously described phylogenies. The pairwise homoplasy index Ö_ù_ marginally detected recombination (P = 0.044) while the max ÷^2^ and NSS did not detect recombination.

## Discussion

Although T3SS are common throughout the Enterobacteriaceae, the discovery of LEE, a notorious T3SS based pathogenicity mechanism, in a non-pathogenic *Salmonella* is surprising. The function of this newly discovered SESS LEE is currently unknown, however as *Salmonella* subspecies *salamae* serovar Sofia is frequently found in association with broiler chickens in Australia [1] and it appears to be non-pathogenic to both humans and chickens [40], [41], it is likely that SESS LEE may play a role in attachment to chicken intestinal mucosa or colonization of chickens. It is clear that the SESS LEE island is a novel version of LEE and not just another T3SS based on both gene content and operon structure. It contains all but five of the LEE genes found in other well-characterized LEE islands (Figure 2). Although key effector genes typical of LEE such as *eae* and *tir* are present, three of the missing genes are secreted effectors (*espG, espF,* and *map*), one is the chaperone for EspF (*cesF*), and the function of last gene, *rorf1*, is unclear. Previous mutational analysis knocked out each of the 41 genes in the *C. rodentium* LEE island then used cell culture and mouse model systems to determine the impact of individual genes on pathogenicity [10]. If the results from these experiments are extended to the SESS LEE, then it would be expected that the missing genes would result in some attenuation of early bacterial colonization of the colon and reduced colonic hyperplasia but the capacity for pedestal formation would not be lost. At this time there is no experimental evidence that *S. enterica* subspecies *salamae* strains from which LEE was derived are capable of forming attaching and effacing lesions but this will be investigated in the future.

In this study the genealogy of the LEE T3SS was determined by BI of a head to tail concatenated, codon guided, gene alignment matrix composed of all of the T3SS genes. A ML analysis was also conducted to demonstrate a similar outcome from an alternative phylogenetic method. Careful consideration must be given to the analytical methodology when determining the genealogy of a genomic island such as LEE which is mobilized by HGT in the context of the detection of recombination by statistical tests. Previous work by Castillo et al. [12] demonstrated incongruence between the phylogenies of some genes in LEE, particularly when comparing secreted effectors to the T3SS encoding genes. This work also suggested that the T3SS genes of LEE shared a common genealogy due to the linkage necessitated by encoding a functional structure. Although a single gene can be used to describe the phylogeny for a species (for the sake of this discussion species refers to any multi-gene genetic unit from a genomic island to an entire organism) it has been demonstrated that phylogenies based upon multiple genes more accurately describe the species phylogeny (reviewed in [42]). A species phylogeny derived from multiple genes is typically calculated by concatenation of multiple genes into a single alignment matrix from which a phylogeny is derived or by congruence of multiple single gene trees. The optimal methodology remains a matter of contention [43]–[45] but there is unlikely to be a “one size fits all” solution. A concatenation method was chosen because the low level of diversity and the detection of recombination would make congruence of single gene phylogenies problematic. Analysis by BI and ML of the T3SS concatenation matrix yielded a tree with high posterior probabilities/bootstrap values for all but one branch (Figure 3). Both of these analyses calculated the phylogeny treating the alignment as a single “supergene” with the BI analysis partitioned by each of the three positions in the codon and ML method using a codon based model. The robust nature of this analysis is supported by results of the ClonalFrame analysis which is essentially a Bayesian analysis partitioned by individual genes which accounts for recombination events. ClonalFrame confirmed the statistical testing for recombination and indicated that recombination was affecting the phylogeny but demonstrated that the impact was not substantial with only branches in clade I altered if recombination was not factored into the calculation (Figure 5).

The single gene phylogenies were performed to permit the addition of an outgroup to the phylogenetic analysis in order to clarify the order of descent. To the extent that it is possible to compare these trees with those in Castillo et al. [12] the phylogenies are similar but the previous work used a different model (Tamara-Nei) with a neighbor joining analysis and included duplicated taxa in all of the trees. It is common when examining multiple genes to observe incongruities and this was the case for the single gene alignments with the *escJ* tree demonstrating a substantially different phylogeny than that observed for the *escV, escN,* and *escF* gene trees (Figure 6). The cause of this incongruity is uncertain and might be attributed to recombination, a variation in the rate of mutation, or insufficient phylogenetically informative data. Although nucleotide compositional bias can be an important factor causing incongruence [46] it seems unlikely in this case given the low level of nucleotide compositional bias determined for the LEE genes (Figure S1). Given the low level of diversity (particularly when calculated without the SESS genes), large number of duplicated taxa removed from the alignments, and the presence of polytomies in the *escN* and *escJ* trees use of the concatenation method to overcome the low density of phylogenetic information in the single gene trees appears to be the optimal method for this data set. Three of the single gene trees support the major branching pattern observed in the midpoint rooted LEE T3SS tree. Therefore, it is reasonable to assume that midpoint rooted tree accurately reflect the order of descent so a putative history for LEE based on the inclusion of the new SESS LEE T3SS region would suggest that the ancestral LEE island moved into the Enterobacteriaceae at some point in the more distant past. This ancestral form then diverged between the lineages that resulted in the current form of LEE in *Salmonella* and a second lineage that diverged between the LEE island found in *Citrobacter* and that found in *Escherichia*. This lineage then diverged between the *E. coli* and *E. albertii* TW07627. The *E. coli* lineage then diverged into two distinct groups that have been described previously [9]. One problem with this suggested genealogy is the tight clustering in clade III of *E. coli* O8:H- 3431-4/86 with *E. albertii* TW07627 which differ from one another across the entire T3SS matrix by only 70 base changes out of a total 13,260 bases (è = 0.00528) in the curated alignment matrix. This level of diversity is greater than that observed for clades II, IV, and V but about half that of clade I. The LEE island in *E. coli* O8:H- 3431-4/86 was identified as being inserted at *pheU* adjacent to *yjdC* and *yjdJ* in a region of the chromosome lacking approximately 10 kb encompassing *lysU, yjdL,* and *cadABC*. Given the level of diversity within all of the clades and the genetic distance between clade III and clades I/II a likely explanation of the origin of LEE in *E. coli* O8:H- 3431-4/86 is that HGT has moved the entire island from *Citrobacter* into *Escherichia*.

The *S. enterica* subspecies *salamae* genomic island described here represents a new lineage of LEE. Castillo et al. [12] postulated from GC content, substitution rates, and phylogenetic analysis that the T3SS of LEE remained linked as a cluster throughout their movement within the Enterobacteriaceae. In addition it was postulated that those LEE genes with higher levels of diversity and greater GC content such as *map, tir, eae, espA, espD, espB,* and *espF* may be more recently acquired portions of the island. Therefore, it was suggested that the LEE island assembly was a complex process and that LEE has not been evolving as a single unit. In light of our study, the SESS LEE region clearly reiterates the conservation of the LEE T3SS as a linked cluster of genes observed by Castillo et al. [12] but it also demonstrates that even the more diverse genes like the secreted effectors were part of an ancestral version of LEE. The mosaicism present in the *Citrobacter/Escherichia* LEE is likely due to recombination that has occurred more recently than the split between *Salmonella* and *Citrobacter/Escherichia*. The conservation of overall operon structure combined with the level of phylogenetic diversity between the SESS LEE island and the previously described LEE islands suggests that the entire LEE island may have moved into the Enterobacteriaceae as a single unit and was then subjected to range of recombination events as it moved through *Citrobacter* and *Escherichia*. This does not preclude previously suggested models for the formation of LEE that begin with a T3SS module followed by the addition of remaining LEE genes. Based on previously known LEE islands, it was logical to conclude that the LEE T3SS might have had a separate history from the secreted effectors and other components. In the context of the LEE island presented here there is less evidence for this hypothesis and the greater diversity observed for the non-T3SS sections of LEE is more likely to be evidence of recent recombination rather than suggestive of the mechanism of initial assembly of the LEE island.

The LEE island has been observed in a limited range of locations in the genome. With the exception of LEE from *C. rodentium,* most other LEE islands are inserted at tRNA genes *pheV, pheU,* or *selC.* It is interesting to note that although *E. albertii* TW07627 LEE is not inserted at a tRNA, it is located 16,003 bases away from *pheU.* Analysis of sequences between and surrounding LEE and *pheU* in the *E. albertii* TW07627 genome, suggests that LEE was inserted at *pheU* then a later transposon insertion has caused an inversion of the 16,003 bp region moving LEE away from the *pheU* and reversing the orientation of LEE compared to other LEE regions inserted at *pheU.* SESS LEE is inserted at *pheV* as observed for *E. coli* strains O103:H2 (strains 12009 and RW1374) and O111:H- 11128. Using adjacent *yqpA* and *pheV* as a point of reference for comparing *E. coli* O103:H2 RW1374 and SESS LEE islands both have a different set of flanking region genes followed by LEE4 and TIR operons in the same orientation. The LEE1, LEE2, and LEE3 operons of SESS LEE have been inverted as a single cassette compared to *E. coli* O103:H2 RW1374. It is possible that the loss of *cesF* and *map* normally present in the junction between the TIR and LEE3 operons may have occurred when this recombination took place. Alternatively, it is possible that this recombination coincided with the addition of these genes to the LEE island. There is insufficient data to speculate on whether *espF, rorf1,* and *espG* were lost from the SESS LEE or inserted after the split between *Salmonella* LEE and LEE from the other genera but the former seems more likely. Conjecture on how or when *espF, espG, cesF, map,* and *rorf1* may have been lost assumes that these genes were present on the ancestral form of LEE and were not acquired at some point after the split between clade V and clades I-IV. Although the preponderance of LEE regions isolated to date have been arranged LEE1, LEE2, LEE3, TIR, and LEE4 the alternative arrangement in the SESS LEE cannot be dismissed as the putative ancestral operon arrangement.

Jores et al. [47] suggested an in-principle classification of LEE islands by their point of insertion. Primarily, this classification scheme was based on LEE being distributed between the *selC* tRNA and *pheV/pheU* tRNA genes. Detailed analysis by Rumer et al. [48] suggested that *pheU* was a likely ancestral insertion point of LEE in *E. coli* with later horizontal transfer to the *pheV.* Although LEE appears to have entered *E. coli* through insertion into the *pheU* site, our phylogeny for LEE complicates any discussion on the ancestral tRNA insertion point of LEE in the Enterobacteriaceae. The two LEE islands closest to the ancestral LEE are derived from *Salmonella* and *C. rodentium.* SESS LEE is inserted at *pheV* and *C. rodentium* is not near *pheU*, *pheV*, or *selC* and is approximately 100,000 bp from any tRNA gene.

This work describes divergent isolates of the SESS LEE from two *S. enterica* subspecies *salamae* serovar Sofia isolates derived from different niches. Future analysis will attempt to examine the distribution of this new lineage in the *salamae* subspecies as well as the other less studied *Salmonella* subspecies. Although neither expression nor in vivo activity has been demonstrated, the existence of the SESS LEE calls into question all of the previous hypotheses related to the origin and genealogy of a key virulence determinant in human pathogens such as EPEC, EHEC, and ATEC. The SESS LEE phylogeny suggests that LEE entered the Enterobacteriaceae by HGT containing both the structural components of the T3SS as well as the associated accessory proteins and effectors. LEE was then subjected to significant recombination particularly within the *E. coli* cells carrying it. The phylogeny of the T3SS component of LEE including the SESS LEE establishes five clades to which the known LEE can be assigned.

## Supporting Information

Figure S1 **Nucleotide composition for all strains subjected to phylogenetic analysis is represented schematically with diagrams generated by SeqVis 1.5.** Analyses for alignment matrices of all LEE region genes, T3SS genes, and the housekeeping genes are shown in panels A, B, and C respectively. Compositional heterogeneity is illustrated by a de Finetti plot of the average nucleotide composition for each strain with A and T nucleotides represented by W. A de Finetti plot is presented for each of the three positions in the codon as indicated.
(TIF)

Figure S2 **Comparison of the SESS genomic island containing LEE with a genomic segment from** ***S. enterica*** **subspecies** ***arizonae*** **.** A segment of the *S. enterica* subspecies *arizonae* genome from the tRNA-Phe (marked in red) at position 3,207,114 to a hypothetical protein terminating near position 3,245,373 of the genomic island is marked by the dark gray arrows. The SESS LEE genomic island is depicted with T3SS genes in green and other LEE genes in blue. SESS flanking region genes are depicted in light gray. Redundant text from the SESS locus tags has been removed to simplify the figure. The full locus tags are presented for selected genes for subspecies *arizonae* genes while the shortened 4 digit code was used for SESS genes as in Figure 1. Gene functions are abbreviated, putative integrase (INT), putative transcriptional regulator (TR), putative secreted effector (SE). Those segments that match in tblastx comparisons are indicated by the connecting lines
(TIF)

Figure S3 **Graph of genetic diversity (θ), ratio of non-synonymous and synonymous changes, and G+C content across the T3SS sequence alignment matrix.** A schematic depicting the extent of each gene in the curated T3SS alignment matrix is presented above plots of genetic diversity (θ) calculated for all sequences in the alignment (blue line) or with sequences from the two *Salmonella* strains removed (red line). Normalized ratio of dN/dS and G+C content calculated with a 50 bp window size.
(TIF)

Table S1 **Accession numbers for housekeeping genes from the** ***Salmonella*** **strains described in this research.**
(DOCX)

## References

[1] Duffy LL, Dykes GA, Fegan N (2012) A review of the ecology, colonization and genetic characterization of *Salmonella enterica* serovar Sofia, a prolific but avirulent poultry serovar in Australia. Food Research International 45: 770–779.

[2] Hensel M (2004) Evolution of pathogenicity islands of *Salmonella enterica* . Int J Med Microbiol 294: 95–102. 15493819 10.1016/j.ijmm.2004.06.025

[3] Andrews-Polymenis HL, Baumler AJ, McCormick BA, Fang FC (2010) Taming the elephant: *Salmonella* biology, pathogenesis, and prevention. Infect Immun 78: 2356–2369. 20385760 10.1128/IAI.00096-10 PMC2876576

[4] Fookes M, Schroeder GN, Langridge GC, Blondel CJ, Mammina C, et al (2011) *Salmonella bongori* provides insights into the evolution of the Salmonellae. PLoS Pathog 7: e1002191. 21876672 10.1371/journal.ppat.1002191 PMC3158058

[5] IMVS (2010) Australian *Salmonella* Reference Centre, 2009 Annual Report.

[6] Croxen MA, Finlay BB (2010) Molecular mechanisms of *Escherichia coli* pathogenicity. Nat Rev Microbiol 8: 26–38. 19966814 10.1038/nrmicro2265

[7] Dean P, Maresca M, Kenny B (2005) EPEC’s weapons of mass subversion. Curr Opin Microbiol 8: 28–34. 15694854 10.1016/j.mib.2004.12.010

[8] Hyma KE, Lacher DW, Nelson AM, Bumbaugh AC, Janda JM, et al (2005) Evolutionary genetics of a new pathogenic *Escherichia* species: *Escherichia albertii* and related *Shigella boydii* strains. J Bacteriol 187: 619–628. 15629933 10.1128/JB.187.2.619-628.2005 PMC543563

[9] Müller D, Benz I, Liebchen A, Gallitz I, Karch H, et al (2009) Comparative analysis of the locus of enterocyte effacement and its flanking regions. Infect Immun 77: 3501–3513. 19506015 10.1128/IAI.00090-09 PMC2715695

[10] Deng W, Puente JL, Gruenheid S, Li Y, Vallance BA, et al (2004) Dissecting virulence: systematic and functional analyses of a pathogenicity island. Proc Natl Acad Sci U S A 101: 3597–3602. 14988506 10.1073/pnas.0400326101 PMC373508

[11] Pallen MJ, Beatson SA, Bailey CM (2005) Bioinformatics analysis of the locus for enterocyte effacement provides novel insights into type-III secretion. BMC Microbiol 5: 9. 15757514 10.1186/1471-2180-5-9 PMC1084347

[12] Castillo A, Eguiarte LE, Souza V (2005) A genomic population genetics analysis of the pathogenic enterocyte effacement island in *Escherichia coli*: the search for the unit of selection. Proc Natl Acad Sci U S A 102: 1542–1547. 15668384 10.1073/pnas.0408633102 PMC547851

[13] Hueck CJ (1998) Type III protein secretion systems in bacterial pathogens of animals and plants. Microbiol Mol Biol Rev 62: 379–433. 9618447 10.1128/mmbr.62.2.379-433.1998 PMC98920

[14] Ross IL, Willmore R, Heuzenroeder MW (2003) A fluorescent amplified fragment length polymorphism study of *Salmonella enterica* serovar Sofia, the major *Salmonella* serovar isolated from chickens in Australia. Int J Med Microbiol 293: 371–375. 14695065 10.1078/1438-4221-00272

[15] Grimont PAD, Weill F-X (2007) Antigenic formulae of the *Salmonella* serovars: WHO Collaborating Centre for Reference and Research on *Salmonella*.

[16] Chia TW, Fegan N, McMeekin TA, Dykes GA (2008) *Salmonella* Sofia differs from other poultry-associated *Salmonella* serovars with respect to cell surface hydrophobicity. J Food Prot 71: 2421–2428. 19244894 10.4315/0362-028x-71.12.2421

[17] Qi J, Zhao F (2011) inGAP-sv: a novel scheme to identify and visualize structural variation from paired end mapping data. Nucleic Acids Res 39: W567–575. 21715388 10.1093/nar/gkr506 PMC3125812

[18] McQuiston JR, Herrera-Leon S, Wertheim BC, Doyle J, Fields PI, et al (2008) Molecular phylogeny of the salmonellae: relationships among *Salmonella* species and subspecies determined from four housekeeping genes and evidence of lateral gene transfer events. J Bacteriol 190: 7060–7067. 18757540 10.1128/JB.01552-07 PMC2580703

[19] Yang J, Nie H, Chen L, Zhang X, Yang F, et al (2007) Revisiting the molecular evolutionary history of *Shigella* spp. J Mol Evol 64: 71–79. 17160643 10.1007/s00239-006-0052-8

[20] Sullivan MJ, Petty NK, Beatson SA (2011) Easyfig: a genome comparison visualiser. Bioinformatics. 10.1093/bioinformatics/btr039 PMC3065679 21278367

[21] Kumar S, Nei M, Dudley J, Tamura K (2008) MEGA: a biologist-centric software for evolutionary analysis of DNA and protein sequences. Brief Bioinform 9: 299–306. 18417537 10.1093/bib/bbn017 PMC2562624

[22] Castresana J (2000) Selection of conserved blocks from multiple alignments for their use in phylogenetic analysis. Mol Biol Evol 17: 540–552. 10742046 10.1093/oxfordjournals.molbev.a026334

[23] Jermiin LS, Ho JW, Lau KW, Jayaswal V (2009) SeqVis: a tool for detecting compositional heterogeneity among aligned nucleotide sequences. Methods Mol Biol 537: 65–91. 19378140 10.1007/978-1-59745-251-9_4

[24] Posada D (2008) jModelTest: phylogenetic model averaging. Mol Biol Evol 25: 1253–1256. 18397919 10.1093/molbev/msn083

[25] Guindon S, Gascuel O (2003) A simple, fast, and accurate algorithm to estimate large phylogenies by maximum likelihood. Syst Biol 52: 696–704. 14530136 10.1080/10635150390235520

[26] Watterson GA (1975) On the number of segregating sites in genetical models without recombination. Theor Popul Biol 7: 256–276. 1145509 10.1016/0040-5809(75)90020-9

[27] Librado P, Rozas J (2009) DnaSP v5: a software for comprehensive analysis of DNA polymorphism data. Bioinformatics 25: 1451–1452. 19346325 10.1093/bioinformatics/btp187

[28] Ronquist F, Huelsenbeck JP (2003) MrBayes 3: Bayesian phylogenetic inference under mixed models. Bioinformatics 19: 1572–1574. 12912839 10.1093/bioinformatics/btg180

[29] Rambaut A, Drummond A (2009) Molecular evolution, phlyogenetics and epidemiology web site, software tool Tracer 1.5. Available: http://tree.bio.ed.ac.uk/software/tracer/. Accessed 2012 Jun 29.

[30] Rambaut A (2010) Molecular evolution, phlyogenetics and epidemiology web site, software tool FigTree 1.3.1. Available: http://tree.bio.ed.ac.uk/software/figtree/. Accessed 2012 Jun 29.

[31] Zwickl DJ (2006) Genetic algorithm approaches for the phylogenetic analysis of large biological sequence datasets under the maximum likelihood criterion: The University of Texas at Austin.

[32] Felsenstein J (1985) Confidence-limits on phylogenies - an approach using the bootstrap. Evolution 39: 783–791. 28561359 10.1111/j.1558-5646.1985.tb00420.x

[33] Sukumaran J, Holder MT SumTrees: Phylogenetic Tree Summarization and Annotation. Available: http://packages.python.org/DendroPy/scripts/sumtrees.html. Accessed 2012 Jul 9.

[34] Bruen TC, Philippe H, Bryant D (2006) A simple and robust statistical test for detecting the presence of recombination. Genetics 172: 2665–2681. 16489234 10.1534/genetics.105.048975 PMC1456386

[35] Smith JM (1992) Analyzing the mosaic structure of genes. J Mol Evol 34: 126–129. 1556748 10.1007/BF00182389

[36] Jakobsen IB, Easteal S (1996) A program for calculating and displaying compatibility matrices as an aid in determining reticulate evolution in molecular sequences. Comput Appl Biosci 12: 291–295. 8902355 10.1093/bioinformatics/12.4.291

[37] Didelot X, Falush D (2007) Inference of bacterial microevolution using multilocus sequence data. Genetics 175: 1251–1266. 17151252 10.1534/genetics.106.063305 PMC1840087

[38] Kosakovsky Pond SL, Frost SD (2005) Not so different after all: a comparison of methods for detecting amino acid sites under selection. Mol Biol Evol 22: 1208–1222. 15703242 10.1093/molbev/msi105

[39] Delport W, Poon AF, Frost SD, Kosakovsky Pond SL (2010) Datamonkey 2010: a suite of phylogenetic analysis tools for evolutionary biology. Bioinformatics 26: 2455–2457. 20671151 10.1093/bioinformatics/btq429 PMC2944195

[40] Harrington CS, Lanser JA, Manning PA, Murray CJ (1991) Epidemiology of Salmonella sofia in Australia. Appl Environ Microbiol 57: 223–227. 1674653 10.1128/aem.57.1.223-227.1991 PMC182689

[41] Heuzenroeder MW, Murray CJ, Dalcin RM, Barton M (2001) Molecular basis of benign colonisation of *Salmonella* Sofia in chickens. A report for the Rural Industries Research and Development Corporation.

[42] Delsuc F, Brinkmann H, Philippe H (2005) Phylogenomics and the reconstruction of the tree of life. Nat Rev Genet 6: 361–375. 15861208 10.1038/nrg1603

[43] Gadagkar SR, Rosenberg MS, Kumar S (2005) Inferring species phylogenies from multiple genes: Concatenated sequence tree versus consensus gene tree. Journal of Experimental Zoology Part B: Molecular and Developmental Evolution 304B: 64–74. 10.1002/jez.b.21026 15593277

[44] Kubatko LS, Degnan JH (2007) Inconsistency of phylogenetic estimates from concatenated data under coalescence. Syst Biol 56: 17–24. 17366134 10.1080/10635150601146041

[45] Rokas A, Williams BL, King N, Carroll SB (2003) Genome-scale approaches to resolving incongruence in molecular phylogenies. Nature 425: 798–804. 14574403 10.1038/nature02053

[46] Jeffroy O, Brinkmann H, Delsuc F, Philippe H (2006) Phylogenomics: the beginning of incongruence? Trends Genet 22: 225–231. 16490279 10.1016/j.tig.2006.02.003

[47] Jores J, Rumer L, Wieler LH (2004) Impact of the locus of enterocyte effacement pathogenicity island on the evolution of pathogenic *Escherichia coli* . Int J Med Microbiol 294: 103–113. 15493820 10.1016/j.ijmm.2004.06.024

[48] Rumer L, Jores J, Kirsch P, Cavignac Y, Zehmke K, et al (2003) Dissemination of *pheU*- and *pheV*-located genomic islands among enteropathogenic (EPEC) and enterohemorrhagic (EHEC) *E. coli* and their possible role in the horizontal transfer of the locus of enterocyte effacement (LEE). International Journal of Medical Microbiology 292: 463–475. 12635929 10.1078/1438-4221-00229

