# Correction: A Genomic Island in *Salmonella enterica* ssp. *salamae* Provides New Insights on the Genealogy of the Locus of Enterocyte Effacement

**DOI:** 10.1371/annotation/bdce3895-9160-4f51-a162-4811b3e6d2e1

**Published:** 2012-11-14

**Authors:** P. Scott Chandry, Simon Gladman, Sean C. Moore, Torsten Seemann, Keith A. Crandall, Narelle Fegan

There were formatting errors in symbols and characters in several locations throughout the text. The corrections are outlined here: http://www.plosone.org/corrections/pone.0041615.cn.tif 

