# Supplementary figures and images for: A Genomic Island in Salmonella enterica ssp. salamae Provides New Insights on the Genealogy of the Locus of Enterocyte Effacement
Source: PLoS One. 2012 Jul 30;7(7):e41615. doi: 10.1371/journal.pone.0041615 (PMC3408504; doi:10.1371/journal.pone.0041615)

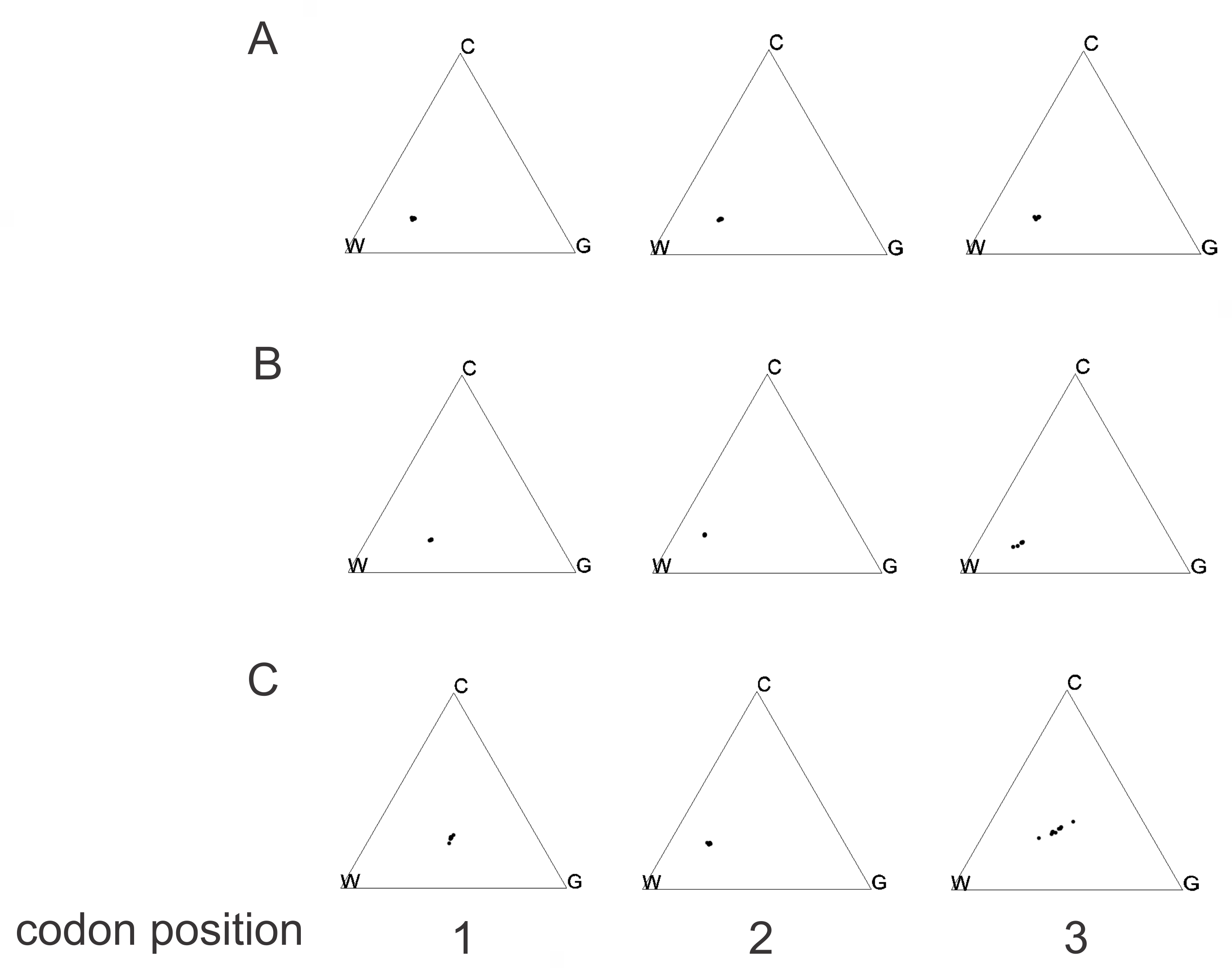

Supplement: Figure S1 — Nucleotide composition for all strains subjected to phylogenetic analysis is represented schematically with diagrams generated by SeqVis 1.5. Analyses for alignment matrices of all LEE region genes, T3SS genes, and the housekeeping genes are shown in panels A, B, and C respectively. Compositional heterogeneity is illustrated by a de Finetti plot of the average nucleotide composition for each strain with A and T nucleotides represented by W. A de Finetti plot is presented for each of the three positions in the codon as indicated. (TIF) [file pone.0041615.s001.tif]

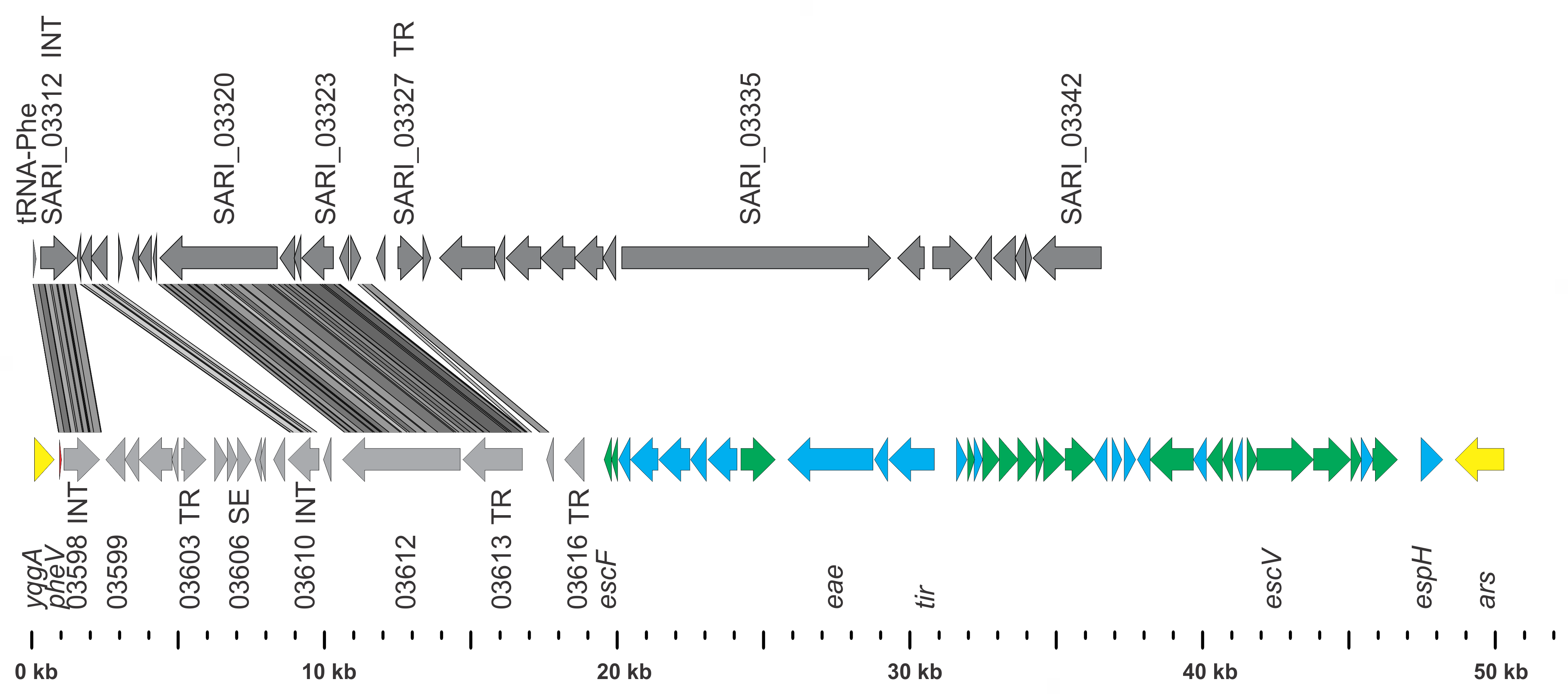

Supplement: Figure S2 — Comparison of the SESS genomic island containing LEE with a genomic segment from S. enterica subspecies arizonae . A segment of the S. enterica subspecies arizonae genome from the tRNA-Phe (marked in red) at position 3,207,114 to a hypothetical protein terminating near position 3,245,373 of the genomic island is marked by the dark gray arrows. The SESS LEE genomic island is depicted with T3SS genes in green and other LEE genes in blue. SESS flanking region genes are depicted in light gray. Redundant text from the SESS locus tags has been removed to simplify the figure. The full locus tags are presented for selected genes for subspecies arizonae genes while the shortened 4 digit code was used for SESS genes as in Figure 1. Gene functions are abbreviated, putative integrase (INT), putative transcriptional regulator (TR), putative secreted effector (SE). Those segments that match in tblastx comparisons are indicated by the connecting lines (TIF) [file pone.0041615.s002.tif]

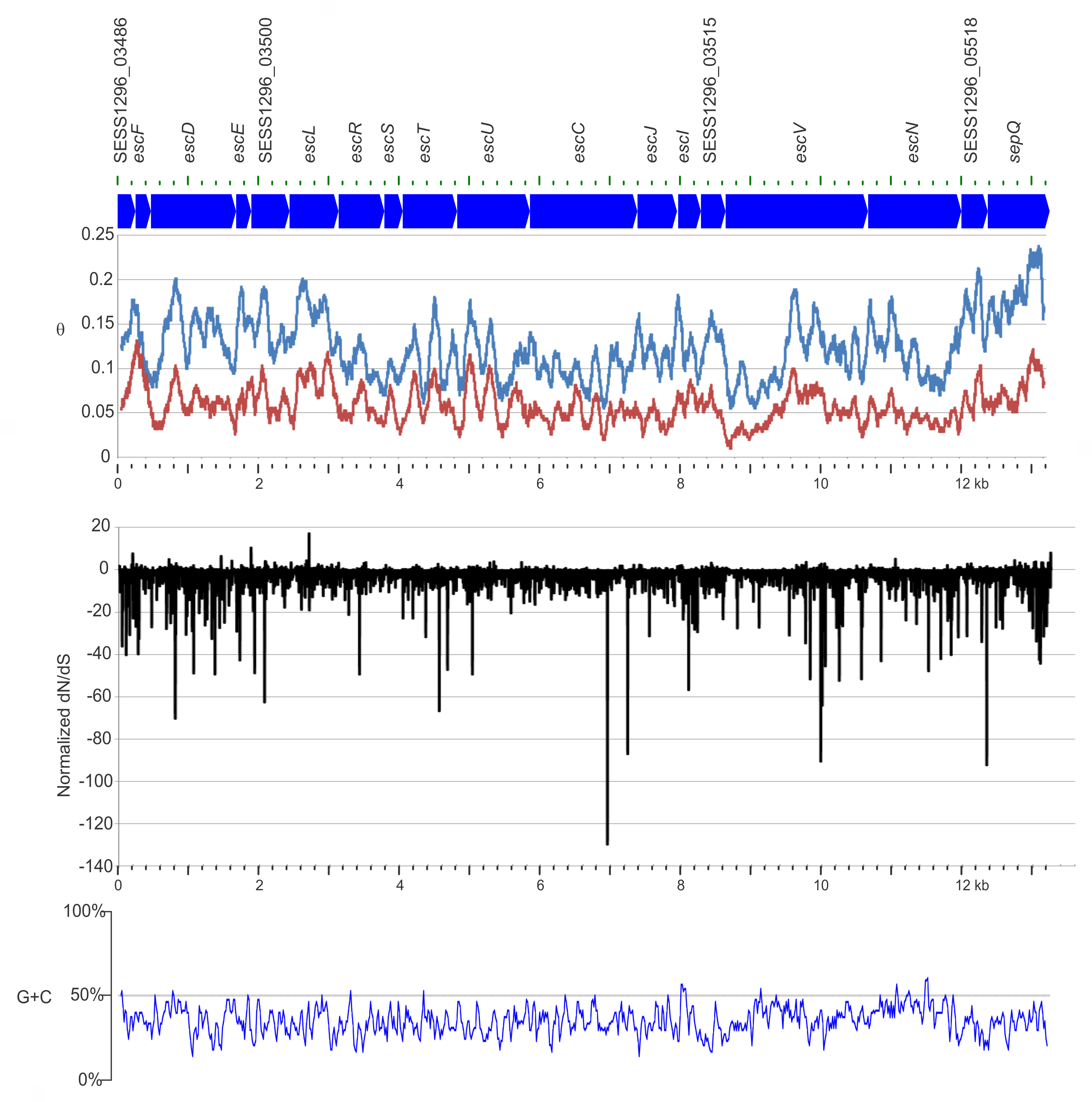

Supplement: Figure S3 — Graph of genetic diversity (θ), ratio of non-synonymous and synonymous changes, and G+C content across the T3SS sequence alignment matrix. A schematic depicting the extent of each gene in the curated T3SS alignment matrix is presented above plots of genetic diversity (θ) calculated for all sequences in the alignment (blue line) or with sequences from the two Salmonella strains removed (red line). Normalized ratio of dN/dS and G+C content calculated with a 50 bp window size. (TIF) [file pone.0041615.s003.tif]
