# Supplementary material for: A Genomic Island in Salmonella enterica ssp. salamae Provides New Insights on the Genealogy of the Locus of Enterocyte Effacement
Source: PLoS One. 2012 Jul 30;7(7):e41615. doi: 10.1371/journal.pone.0041615 (PMC3408504; doi:10.1371/journal.pone.0041615)
Supplement: Table S1 — Accession numbers for housekeeping genes from the Salmonella strains described in this research. (DOCX) [file pone.0041615.s004.docx]

Table S1. Accession numbers for housekeeping genes from the *Salmonella* strains described in this research.

| Gene | S1296 Accession Num. | S1635 Accession Num. |
| --- | --- | --- |
| *accD* | JQ747524 | JQ747541 |
| *dcuA* | JQ747525 | JQ747542 |
| *galK* | JQ747526 | JQ747543 |
| *hemC* | JQ747527 | JQ747544 |
| *ilvE* | JQ747528 | JQ747545 |
| *ksgA* | JQ747529 | JQ747546 |
| *murD* | JQ747530 | JQ747547 |
| *oppB* | JQ747531 | JQ747548 |
| *pabB* | JQ747532 | JQ747549 |
| *pntB* | JQ747533 | JQ747550 |
| *polB* | JQ747534 | JQ747551 |
| *purB* | JX026952 | JX026953 |
| *rnc* | JQ747535 | JQ747552 |
| *rpoN* | JQ747536 | JQ747553 |
| *tesB* | JQ747537 | JQ747554 |
| *thrB* | JQ747538 | JQ747555 |
| *trpB* | JQ747539 | JQ747556 |
